# Program ACTIVE II: Design and Methods for a Multi-Center Community-Based Depression Treatment for Rural and Urban Adults with Type 2 Diabetes

**DOI:** 10.16966/2380-5544.108

**Published:** 2015-08-05

**Authors:** Mary de Groot, Jay Shubrook, Frank Schwartz, W. Guyton Hornsby, Yegan Pillay, Chandan Saha

**Affiliations:** 1 Indiana University School of Medicine, USA; 2 Touro University School of Medicine, USA; 3 Ohio University Heritage College of Osteopathic Medicine, USA; 4 West Virginia University School of Medicine, USA; 5 Ohio University, USA

## Abstract

**Objective:**

Depression affects one in four adults with type 2 diabetes (T2DM) and is associated with worsened diabetes complications, increased health care costs and early mortality. Rural and low-income urban areas, including the Appalachian region, represent an epicenter of the T2DM epidemic. Program ACTIVE II is a comparative effectiveness treatment trial designed to test whether a combination of cognitive behavioral therapy (CBT) and community-based exercise (EXER) will offer greater improvements in diabetes and depression outcomes compared to individual treatment approaches and usual care (UC). The secondary aims are to assess changes in cardiovascular risk factors across groups and to conduct a cost-effectiveness analysis of predicted incidence of cardiovascular complications across groups.

**Methods:**

The study is a 2-by-2 factorial randomized controlled trial consisting of 4 treatment groups: CBT alone, EXER alone, combination of CBT and EXER, and UC. Adults with T2DM for > 1 year and who meet DSM-IVTR criteria for Major Depressive Disorder (MDD) are eligible to participate at two rural Appalachian sites (southeastern Ohio and West Virginia) and one urban site (Indianapolis). This type II behavioral translation study uses a community-engaged research (CEnR) approach by incorporating community fitness centers and mental health practices as interventionists.

**Conclusions:**

This is the first study to evaluate the comparative effectiveness of combined CBT and exercise in the treatment of depression using community-based intervention delivery. This approach may serve as a national model for expanding depression treatment for patients with T2DM.

## Introduction

Nearly one in 10 Americans has diabetes and current projections estimate diabetes will affect one in three Americans born in the year 2000 [1] resulting in annual T2DM-related costs of $256 billion [2]. Low-income urban and rural areas such as the Appalachian region bear a disproportionate burden of the national T2DM epidemic [3]. In West Virginia (WV), the statewide age-adjustment prevalence of diagnosed T2DM is 10.4% [4] while the average prevalence rate of T2DM in rural Appalachian Ohio (OH) counties is 11.3% [5] exceeding the national average of 9.3% [1].The Appalachian region also bears a disproportionate burden of major depressive disorder (MDD; 8.2%) compared to non-Appalachian areas (7.6%), with higher rates found in central Appalachian states (10.6%) such as WV and OH than northern or southern states [6].

Barriers to care for T2DM and MDD in low-income urban and rural areas such as central Appalachia are considerable and similar. Decreased access to health and mental health care providers, lack of public transportation and rising costs for private transportation (e.g. gas prices) in the context of geographic isolation pose significant barriers to consistent and effective health and mental health care [7–8]. Patients with T2DM have been found to be two times more likely to experience depressive symptoms than their non-diabetic peers with one in four patients reporting clinically significant depressive symptoms and 11.4% meeting criteria for MDD [9].

Depressive symptoms have been shown to be associated with worsened blood glucose levels [10] and T2DM complications [11]. In addition, significant functional and financial costs are associated with depression and T2DM including decreased adherence to diabetes care regimens[12], increased functional disability [13], increased health care costs without resulting improvements in depression or diabetes outcomes [14], decreased quality of life [15–16]and earlier mortality attributable to all causes (17–18).

Few controlled treatment trials have been conducted to treat depression and T2DM [19–23]. Only three have evaluated behavioral treatment strategies in this population. In a meta-analysis of 29 randomized controlled trials evaluating cognitive behavioral therapy (CBT) treatment for MDD across a continuum of disease states, a robust effect size (−.83) documented the efficacy of this approach [24]. In a study of 51 patients (meanage53+10years;60%female)diagnosedwithT2DM(meanduration 8 years) and MDD randomly assigned to a 10-week individualized CBT or control condition without medication intervention, Lustman found that patients receiving CBT were 3 times more likely to experience depression remission at post-treatment assessment than controls [19]. Although no group differences in A1c levels were found at post-treatment assessment (adjusted for baseline values), patients in the CBT condition showed improvement in A1c at the 6-month follow-up assessment compared to controls [19]. Treatment responsiveness appeared to be associated with severity of depression and A1c at baseline [19,25].

In the Pathways Study, problem-solving therapy (a variation of CBT) was integrated within the primary care setting to treat depression in adults with T2DM [23]. Participants randomized to a stepped-care problem-solving therapy intervention reported higher levels of treatment exposure, satisfaction with care and improved depression outcomes compared to patients in the usual care group. In this study, improvements in glycemic control were not observed immediately following care or at 6- or 12-month follow-up assessments [23].Taken together; these studies demonstrate the efficacy of CBT in treating depression in adults with T2DMwith mixed results associated with changes in glycemic control.

Exercise has the potential to directly improve glycemic outcomes in the context of MDD treatment and has been shown to be an efficacious treatment for clinical depression among adults without T2DM [26–27]. In a meta-analysis of 37 exercise intervention trials in clinically depressed samples, Craft & Landers [28]found a large overall effect size (−.72) in the reduction of clinical depression with the greatest impact found in samples with moderate-to-severe levels of depression. Exercise interventions of 9 to12 weeks showed significantly greater impact on depression outcomes, but no differences were observed in the types of exercise used in treatment protocols. Exercise interventions have also been shown to improve glycemic control in patients with T2DM [29–34].

To date, only one randomized controlled trial (RCT) has examined the impact of exercise on depression in adults with type 2 diabetes. Piette and colleagues randomized 291 adult patients with T2DM and depressive symptoms (mild to severe) to 12 weeks of manualized telephone-based CBT (with 9 monthly booster sessions) with a walking programor enhanced UC [35]. At 12-month follow-up, 58% of participants in the intervention group had depressive symptoms fall below the mild threshold. Number of steps was higher and systolic blood pressure was lower in the intervention group at follow-up. No changes were observed in A1c at the 12-month follow-up assessment [35]. The telephone-based CBT and walking interventions were combined and could not be evaluated for depression and A1c outcomes separately.

To date, the existing literature has utilized traditional randomized controlled trials that make use of research or health care personnel to implement the interventions. No studies have evaluated the comparative effectiveness of CBT and exercise by implementing these interventions with community fitness and mental health providers using a community-engaged research (CEnR) approach. The challenge for health care providers and communities is to create depression treatment programs for T2DM patients that will reach the largest number of people at the lowest possible cost and burden to health care organizations. Program ACTIVE II has been designed to create a model program of community-based depression treatment using a CEnR design that may be disseminated and adopted nationally. Central to this model is the assumption that effective depression treatment requires multiple avenues of access beyond the formal health care system and multiple approaches to depression treatment such as CBT and exercise. Program ACTIVE II has been designed to simultaneously achieve two overarching goals: 1) to test the comparative effectiveness of the cognitive behavioral therapy (CBT) and community-based exercise (EXER) to usual care (UC); and 2) to develop a sustainable program that may be used as a scalable model for T2DM and depression treatment for national dissemination. The research team has partnered with community mental health providers and exercise facilities to implement the intervention. This paper reports on the innovative design and methodology of this large-scale multi-site trial.

## Research Design and Methods

Program ACTIVE II is a 2-by-2 factorial repeated measures RCT design. The study is a multi-center trial in 3 states designed to maximize generalizability of the findings across high risk groups in both urban and rural areas to assess outcomes and to build the infrastructure necessary to achieve a sustainable program. Program ACTIVE II uses a community-engaged research (CEnR) approach in which community organizations are engaged in all aspects of the study: recruitment, intervention implementation, and dissemination of findings. This approach confers additional validity to the study outcomes by examining the effectiveness of the intervention in the sites where they are ultimately designed to take place and demonstrated stakeholder commitment to the intervention which serves as a foundation for the ultimate adoption of the Program ACTIVE intervention beyond the period of federal funding.

The purpose of Program ACTIVE II is to address two primary aims: 1) to compare changes in glycemic control across individual intervention groups to usual care (UC) at post-intervention (POST) and 6- and 12-month follow-up assessments; and 2) to compare changes in MDD outcomes across individual intervention groups to UCat POST and 6- and 12-month follow-up assessments. The study will also address two secondary aims: 1) to compare changes in cardiovascular risk factors (e.g. LDL cholesterol) across individual groups to UC at each of the 3 time points; and 2) to calculate the cost-effectiveness of each treatment arm in terms of predicted incidence of cardiovascular complications over a 10-year period.

### Sample size

Based on our pilot study [36] and a meta-analysis by Boule et al., we used a conservative assumption of a mean (SD) difference of 0.6% (1.4%) in improving A1c between the UC and exercise groups at post intervention [29].Using a sample size of 43 in each of the four treatment groups will have 80% power to detect the main effect of exercise. However, we will recruit 54 per group assuming a 20% dropout rate.

In estimating the sample size to detect changes in MDD outcomes, we assumed 60% and 30% remission rates for the CBT and UC groups, respectively, at post intervention [19]. Using a sample size of 43 in each of the four treatment groups will have 97% power to detect the main effect of CBT.

### Randomization

A site-specific computer generated randomization list was used to randomly assign study subjects to one of the four treatment groups. A block size of four was used to ensure balance in number of subjects recruited in four groups after recruiting every four subjects.

### Characteristics

#### Inclusion/Exclusion Criteria

Inclusion criteria: age 18 or older, ambulatory status, diagnosis of T2DM for one year duration or longer, major depression lasting 2 weeks or longer with no evidence of psychotic symptoms. Medical exclusion criteria include: history of diabetic ketoacidosis (DKA), continuous insulin therapy since T2DM diagnosis, stage 2 hypertension as defined by JNC VII, recent cardiac events (e.g. unstable angina, diagnosed angina, PTCA, any cardiac intervention for CAD or tachydysrhythmias in the past 6 months), laser surgery for proliferative retinopathy in the past 6 months, history of stroke, lower limb amputation, asensory peripheral neuropathy, aortic stenosis or other severe valvular heart disease, atrial fibrillation, severe COPD (e.g., basal oxygen), class III or IV heart failure or medical instability. Psychiatric exclusion criteria include: active suicidal ideation or a history of suicide attempt, bipolar depression or history of psychotic disorder and current substance abuse or dependence disorder. Participants who are currently prescribed antidepressant medications for 6 weeks or longer and who meet diagnostic criteria for major depression without psychotic features are included.

Participants who report the use of a current antidepressant medication for 5 weeks or less are excluded or deferred for later screening after the 6-week period. Participants who are currently receiving psychotherapy from a mental health provider for MDD are excluded. Participants who are currently receiving only medication management from a psychiatrist are included. Respondents who meet eligibility criteria are invited to participate in the baseline screening assessment and referred to their local site PI.

### Recruitment

**Figure 1:**
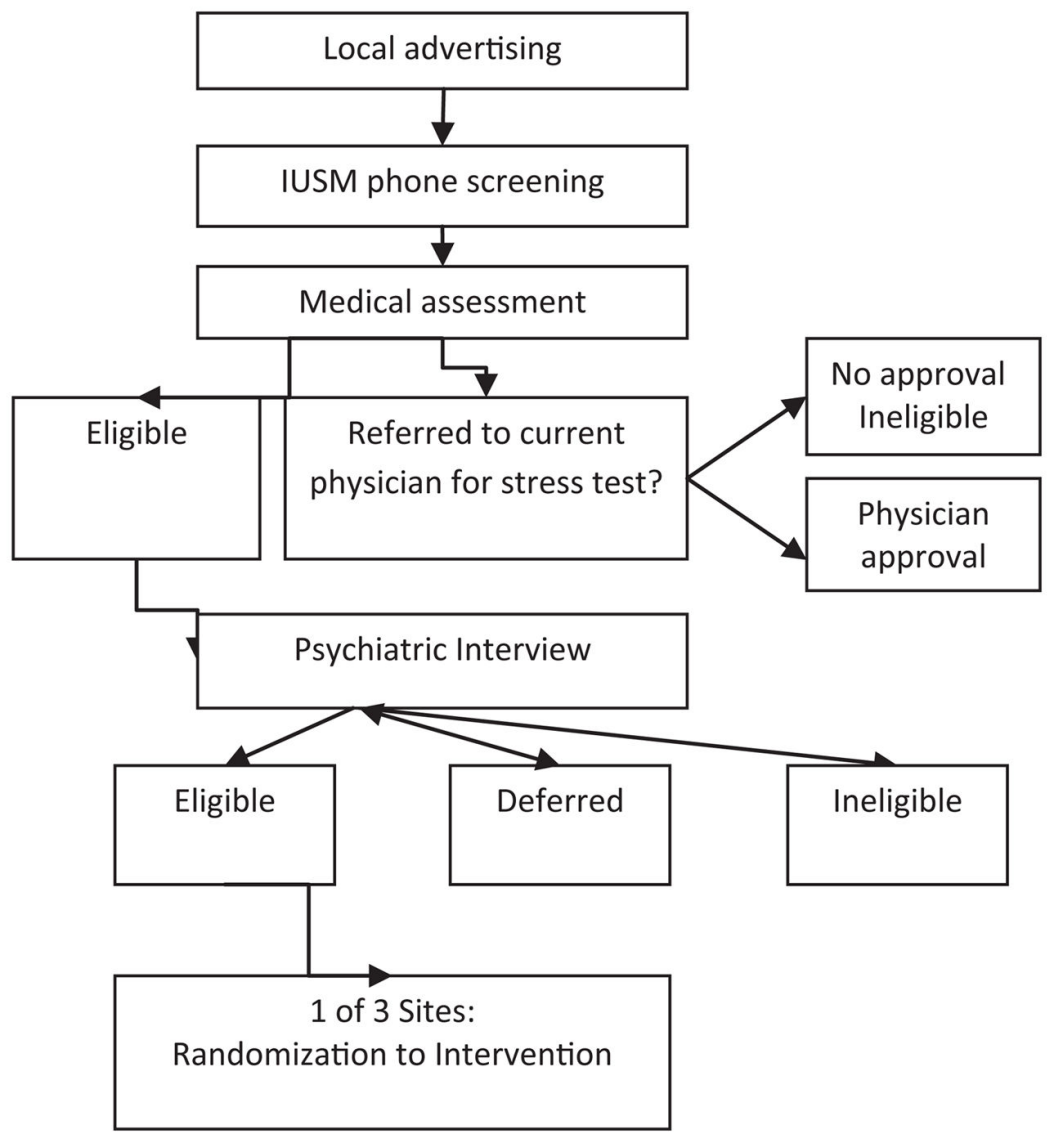
Recruitment Flow Chart Separate randomization lists were generated by the study statistician for use by each study site.

Participants are recruited from communities served by the 3 intervention areas: Indianapolis, southeastern Ohio/western West Virginia and north central West Virginia. Advertising takes place via physician offices, newspapers, radio stations, community centers, partnering community organizations and community events. In addition, patients of partner medical practices are contacted by phone to receive information about the study and to inquire about interest and eligibility (IU and WV). The recruitment flow chart is shown in Figure 1 (below).Individuals are screened by telephone by trained study staff to determine initial eligibility. Once eligible via phone screen, participants are scheduled for a baseline assessment visit where the informed consent takes place. Medical and psychiatric data from the baseline assessment are reviewed by the study or site medical directors (JS. FS, KF, KM), exercise physiologist (GH) and PI (MdG) as a group to determine eligibility for study enrollment. Eligible participants are then randomly assigned to one of the four groups: CBT, EXER, CBT+EXER or UC.

### Assessment procedures

Psychological, behavioral and physiologic measures are administered at each assessment period: baseline, post-intervention, 6- and 12-month follow-up with the exception of demographic characteristics (baseline only). Measures are shown in Table 1.

At the beginning of the baseline assessment visit, individuals are consented to participate in the study by the Site PI or project coordinator. Baseline assessment includes the medical history interview, blood draws, anthropometric measurements and fitness assessment. Participants are given a pedometer, food and activity diaries and questionnaires to complete following the baseline assessment. Participants complete psychosocial measures at home (returned by mail) and complete a SCID Axis I interview by phone. The core investigator team, site PIs and project coordinators conduct weekly teleconferences to review all data following baseline assessment. Participants who meet all study criteria are randomized to one of the four intervention groups, scheduled for a free nutritional education program and their first intervention session (CBT, EXER or CBT+EXER) the following week.

### Interventions

The active interventions in Program ACTIVE II are 12-weeks of community-based exercise and 10 individual sessions of CBT. In order to facilitate diabetes education and to offer an incentive to UC participants, *Dining with Diabetes* nutrition classes are provided to participants in all arms. Based on prior studies, nutrition education is not expected to influence depression outcomes [19].

#### CBT Intervention

CBT has gained wide acceptance as an efficacious intervention approach for the treatment of depression [50]. CBT posits that cognitions, emotions and behaviors are interwoven and mutually reinforcing in the depressed patient [50]. Treatment involves the identification and reframing of “automatic thoughts” (i.e., cognitive biases) that work in the service of depressogenic core cognitive beliefs [50]. Cognitive biases and core beliefs are empirically tested and restructured. Behavioral techniques, such as increasing daily activity and development and interaction with social support networks are interwoven into CBT therapy [50]. The use of cognitive and behavioral tools modeled in therapy and generalized by the participant through take home activities provides participants with an opportunity to generalize skills to situations beyond the therapeutic relationship.

CBT sessions are conducted by trained licensed mental health providers currently in practice in their respective communities. They represent the range of practice environments from individual private practitioners to community mental health centers. Participants receive 10 sessions of CBT using the manualized approach based on Beck’s model of cognitive therapy [50].Sessions are scheduled weekly over the course of the 12-week period. In light of the large array of skills that are possible to include in the CBT framework, selected goals have been targeted for our brief therapy format (presentation of CBT model; thought records, cognitive distortions, counterarguments, cognitive reframing, automatic thoughts, core beliefs, and relapse prevention). CBT therapists receive training in CBT and manualized training approach from the study team.

#### Exercise Intervention

The exercise protocol is a community-based exercise intervention based on the aerobic exercise goals adapted from the Lifestyle Balance behavior arm of the DPP [44]. Partner exercise facilities represent a range of fitness facilities including physical therapy practices, community centers and for-profit and non-profit fitness organizations (e.g. YMCA). Trained instructors at each facility provide 6 classes of monitored instruction on exercise to participants to meet heart rate and activity-level goals. Membership to the centers is provided to participants free of cost throughout the 12-week intervention period. Passes to fitness facilities and costs for time spent with the fitness trainer are purchased by the grant to increase access to exercise for the duration of the protocol. These costs replicate costs of the program to community members of an independent program.

Exercise goals are adapted to accommodate the physical and medical restrictions of an older-adult T2DM population. Exercise prescriptions are based on the results obtained from the baseline 6MWT. Participants are given exercise goals of performing 150 minutes per week of moderate activity at 40- <60% of heart rate reserve (HRR), comparable to a Rating of Perceived Exertion of 11–13. These goals may be modified based on results of the 6 Minute Walk Test (6MWT). Due to high rates of sedentary behaviors in this population, physical activity goals are increased in a graduated fashion during Weeks 1–3, beginning with 100 minutes of weekly aerobic exercise to 150 minutes of total aerobic weekly exercise by Weeks 4–12 [47]. Intensity of activity begins at 40% in Week 1 and progresses as tolerated, but continues to remain below 60% of HRR.

In order to provide participants with the necessary training to begin a safe exercise program, exercise classes are taught by trained fitness instructors at each exercise site in Weeks 1–4 and again in Weeks 6 and 8. In Week 1, participants are introduced to the proper use of exercise equipment and the exercise prescriptions. During these sessions, participants are monitored for 50-minutes of exercise by the exercise fitness instructor. Participants are trained to exercise in a manner consistent with ACSM recommendations including 10 minutes of pre-activity (warm-up and stretching), 30 minutes of active exercise (endurance), and 10 minutes of post-activity (cool down, recovery) [47,51]. Participants receive feedback throughout the 50-minute session on their intensity for each given activity. Heart rate and blood pressure are measured at rest, at peak activity level, and following recovery. Participants are trained to utilize the Borg Scale [52] during their activity to monitor exercise intensity.

In Weeks 2–4, participants are given personalized instruction on exercise modes available including walking on a track, use of treadmills and stationary recumbent/upright ergometers consistent with the equipment of each fitness location. In weeks 6 and 8, participants attend an in-person booster session at their local exercise center to re-establish the appropriate exercise intensity and assess exercise technique. Exercise prescriptions may be adjusted at this time based on the information obtained about exercise intensity. At each class, a chapter from the Program ACTIVE II Exercise Manual is provided to participants. Adapted from the DPP Lifestyle Balance intervention materials, the manual is designed to address psychological barriers associated with physical activity (e.g. social support, motivation, behavioral goals).

Participants are asked to complete weekly exercise diaries and to record number of steps measured using pedometers provided by the study (Weeks 1–12) on paper or in electronic form through the website. Participants are contacted bi-weekly by a member of the study team to review information from activity diaries and pedometers and to evaluate any exercise-related medical concerns including: hyperglycemia, hypoglycemia, joint pain, back pain, angina, lightheadedness, and symptoms of hypotension. Data from participants who report medical symptoms resulting from exercise will be shared with the Medical Director for the respective site. The development of severe adverse events is communicated to the site Medical Director, Data Safety and Monitoring Panel and hosting IRB offices for consultation. SMBG data is downloaded electronically to examine significant changes in daily blood sugars as a result of exercise. Participants who show increased instances of hyper- or hypoglycemia are contacted by the project coordinator and advised to contact their primary care provider for assessment of medications. Participants are provided with electronic (via website) and paper (as needed) supplemental adherence materials (i.e. toolkit) such as walking maps, self-care information, and games to promote adherence.

#### Nutrition Intervention

In order to evaluate the effectiveness of the target interventions in the context of T2DM treatment best practices, participants in all 4 groups are provided classes in the Dining with Diabetes (DWD) program through The Ohio State University, West Virginia University Extension or Purdue University Marion County Extension.

### Statistical analyses

The intention-to-treat principle will be used for all analyses. Differences in participants’ baseline characteristics among 4 groups will be evaluated by ANOVA or the nonparametric Kruskal-Wallis test for continuous outcomes and by Chi-square test for categorical outcomes. A mixed-model analysis of variance including site, treatment group, time, gender, race, age, baseline value of A1c will be used to assess the effects of exercise on A1c at post-intervention, 6- and 12-month. Participants will be treated as a random effect and a un-structure covariance matrix will be used. The dichotomous primary outcome, remission in depression, will be analyzed by a non-linear mixed-effects model and will adjust for potential confounder’s gender, race, age and baseline depression score. In each of the above two models, an interaction effect between time and treatment group will be assessed first. If there is no interaction effect, the overall treatment difference will be assessed. Otherwise, treatment difference will be assessed at each time point. The model will be fitted by the SAS procedure NLMIXED.

## Secondary Outcomes

### Functional exercise status and cardiovascular risk factors

A repeated measures ANCOVA will be conducted to assess changes in highest HR during the 6 minute walk test, distance achieved, LDL-C, HDL-C, triglycerides, and resting blood pressure from baseline to POST and baseline to the 6- and 12-month assessments.

### Cost-effectiveness (CE) analyses

A Markov decision model of type 2 diabetes progression to complications will be constructed using cost data collected at baseline, post-intervention, 6 and 12 months of follow-up. Short- and long-term effects of the intervention can be estimated by including time horizons 3, 10, 20 and lifetime in the Markov model. All costs will be adjusted to the year 2007 using the US Consumer Price Index.

### Markov models

We will develop a Markov decision model with sensitivity analyses to estimate the Incremental Cost-Effectiveness ratio (ICER) of the intervention compared to UC as implemented in Program ACTIVE II. An ICER is computed by the ratio of ΔC/ΔE where ΔC represents the change in cost due to the intervention compared with UC and ΔE represents the change in health benefits due to the intervention compared with UC. We will use TreeAge Pro Suite 2009 software (TreeAge Software, Williamstown, MA). The model will directly incorporate intervention effectiveness and cost data as well as event probabilities from the RCT to estimate life expectancy, quality-adjusted life-expectancy (expressed as QALYs), clinical outcomes (diabetes complications), and direct medical and nonmedical costs associated with the interventions and UC.

## Data Safety and Monitoring Panel

A Data Safety and Monitoring Panel (DSMP) were created to provide expert consultation in the primary disciplines involved in the content of the study: psychology, medicine, and exercise physiology. These consultants provide input on issues such as metabolic or physiological changes in participants during participation, mental status of participants, and implementation of the exercise protocol throughout Program ACTIVE II. Members of the panel are available for consultation for issues of research ethics, clinical care, and human subject’s protection. Members of the panel are independent from the research team to ensure objectivity in the treatment of participants.

Data integrity is reviewed by the Core Investigators in consultation with Dr. Chandan Saha of the IUSM Department of Biostatistics. The Core Investigators meet monthly by phone to discuss issues related to recruitment, implementation of the intervention, participant safety, and to monitor data trends (e.g., adherence, missing data.

## Conclusions

T2DM and co-morbid depression represents a growing challenge to patients and health care systems as the prevalence of both disorders rises but access to treatment remains limited. In this study, we are investigating the comparative effectiveness of a combination of CBT and exercise using a CErN approach in which community fitness and mental health partners provide depression treatment to study participants. This approach is novel because it is the first study to examine the comparative effectiveness of two behavioral treatment approaches to depression and T2DM. Moreover, it leverages community resources such as fitness centers, community centers and community mental health professionals that already exist in rural and low-income urban communities to serve as treatment partners to health care centers. At the same time, it expands knowledge of diabetes treatment issues to these community providers thereby expanding continuity of care for participants.

Data from this study will provide the empirical foundation for community stakeholders to evaluate the value of this program in terms of health and mental health outcomes in their communities and to identify the ways that this may become a sustainable treatment resource for communities beyond the period of federal funding. Lessons learned from this study will also inform the dissemination of this intervention as a model that may be scaled to the national level to alleviate the burden and costs with this important set of disorders.

## Supplementary Material

1

## Figures and Tables

**Table 1:** Administration of Assessment Measures in Program ACTIVE

Measures	Screening	Baseline	POST	6- and 12- Mo. Follow-Up	Outcome Variable	Covariate	Clinical Monitoring Variable
**Psychosocial**							
Demographic Characteristics		**X**				X	
Structured Clinical Interview for the DSM-IVTR (SCID Axis I Disorders)	**X (Screener)**	**X**	**X**	**X**	Primary		
		**Lifetime**	**Current**	**Current**			
Beck Depression Inventory (BDI)		**X**	**X**	**X**	Primary		
Diabetes Quality of Life (DQOL)		**X**	**X**	**X**	Secondary		
SF-36 Quality of Life Measure		**X**	**X**	**X**	Secondary		
Chronic Illness Resource Survey (CIRS)		**X**	**X**	**X**	Secondary		
**Behavioral**							
Physical Activity Diary (1 week)		**X**	**X**	**X**			**X**
Pedometer (1 week)		**X**	**X**	**X**			**X**
**Physiologic**							
Glycated Hemoglobin (A1c)		**X**	**X**	**X**	Primary		
Blood lipid profile (HDL-C; triglycerides)		**X**	**X**	**X**	Secondary		
Self-Monitored Blood Glucose (SMBG)		**X**	**X**	**X**			**X**
Medical History Interview	**X**						**X**
Medical Status Review			**X**	**X**			**X**
6-Minute Walk Test		**X**	**X**	**X**	Secondary		
Height		**X**	**X**	**X**		**X**	
Weight		**X**	**X**	**X**		**X**	
Waist/Hip Girth		**X**	**X**	**X**			
Blood Pressure		**X**	**X**	**X**	Secondary		**X**
Resting Heart Rate/Pulse		**X**	**X**	**X**			**X**
Perceived Exertion (Borg rating)		**X**	**X**	**X**			**X**
