# Supplementary material for: Program ACTIVE II: Design and Methods for a Multi-Center Community-Based Depression Treatment for Rural and Urban Adults with Type 2 Diabetes
Source: J Diabetes Res Ther. Author manuscript; Available in PMC 2016 Aug 4. (PMC4974010; doi:10.16966/2380-5544.108)
Supplement: 1 [file NIHMS730501-supplement-1.pdf]

SciForschen
Open HUB for Scientific Research
ISSN 2380-5544
Journal of Diabetes Research and Therapy
Review Article
Volume: 1.2
Open Access

# Program ACTIVE II: Design and Methods for a Multi-Center Community-Based Depression Treatment for Rural and Urban Adults with Type 2 Diabetes

**Mary de Groot[1]*, Jay Shubrook[2], Frank Schwartz[3], W. Guyton Hornsby Jr[4], Yegan Pillay[5], Chandan Saha[1]**

[1]*Indiana University School of Medicine, USA*
[2]*Touro University School of Medicine, USA*
[3]*Ohio University Heritage College of Osteopathic Medicine, USA*
[4]*West Virginia University School of Medicine, USA*
[5]*Ohio University, USA*

**Received date:** 18 June 2015; **Accepted date:** 31 July 2015; **Published date:** 5 August 2015.

**Copyright:** © 2015 de Groot M, et al. This is an open-access article distributed under the terms of the Creative Commons Attribution License, which permits unrestricted use, distribution, and reproduction in any medium, provided the original author and source are credited.

***Corresponding author:** Mary de Groot, Ph.D., Associate Professor, Indiana University School of Medicine, 410 W. 10th St., Suite 1140, Indianapolis, IN 46202, USA, Tel: (317) 278-1965; Fax: (317) 278-1750; E-mail: **E-mail:** mdegroot@iu.edu

## Appendix I

### Program ACTIVE Measures

Psychological, behavioral and physiologic measures are administered at each assessment period: baseline, post-intervention, 6- and 12-month follow-up with the exception of demographic characteristics (baseline only).

*Structured Clinical Interview for the DSM-IVTR (SCID)* is a semi-structured diagnostic interview schedule used to assess lifetime and current diagnoses of Axis I psychiatric disorders using the American Psychiatric Association DSM-IVTR. The SCID represents the gold standard of diagnostic assessment for psychiatric disorders permitting interviewers to distinguish symptoms attributable to MDD from diabetes and other medical or psychiatric co-morbidities. Symptoms are then evaluated for co-occurrence in time (syndrome), presence/absence of clinical impairment (disorder) and differential diagnosis. Interviewers are trained to reliability on practice interviews to establish consistency in clinical thresholds for symptom presence/absence. The SCID has been shown to have adequate inter-rater (*kappa* = .61- .68) and test-re-test reliabilities (*r* = .64-.69 for major depression) and validity [37, 38]. The PI is expert in the use and training of the SCID. Interviewers complete 15 hours of didactic training and 5 taped practice interviews. A case conference process is used to achieve consensus ratings of diagnoses and to monitor reliability, quality assurance of the phone interviews and drift. Presence/absence of MDD diagnosis is the primary depression outcome.

*Beck Depression Inventory (BDI-II)*is a 21-item self-administered questionnaire used to assess symptoms of depression [39]. The BDI-II has been shown to have excellent test-retest reliability and validity when used in general populations [39] as well for use with T2DM samples [40]. The BDI is administered at each assessment time point for all participants and weekly during CBT treatment in the CBT and CBT+EXER arms.

*SF-12 Quality of Life Measure (SF-12)* is a 12-item self-administered questionnaire used to assess general quality of life [41]. Internal consistency in adult samples has been found to be r = .81-.88. The measure has shown acceptable validity in T2DM samples [42] . The MCS and PCS subscales as well as the 6D utility will be used to calculate quality-adjusted life years in the cost effectiveness analyses.

*God in Leisure-Time Physical Activity Scale* is a 2-item scale used to measure current physical activity level [43]. Participants are asked to estimate weekly participation in strenuous, moderate, and mild activity and rate the frequency of routine exercise behaviors (e.g. often, sometimes, and never/rarely).The instrument has been found to possess acceptable concurrent validity and reliability (test-retest correlations ranged from $r$ = 0.62 for item 1, and $r$ = 0.69 for item 2).

*Demographic characteristics* are collected via a self-administered questionnaire. Data collected includes age, ethnicity, marital status, income, educational status, work status, and health insurance status.

*Physical Activity Diaries* adapted from the DPP Lifestyle Balance Intervention condition [44]are used to record physical activity for a one-week period at each assessment. Total duration of physical activity is recorded weekly as a measure of adherence for all participants randomized to Exercise alone or CBT+EXER conditions during the intervention period.

*Number of Steps* are measured using the Yamax Digiwalker SW200 pedometer (Optimal Health Products, Inc.) during a one-week period at each assessment. The measure will be used to assess caloric expenditure through the change in steps [60] and an estimate of kilocalories per minute energy expenditure [45].Data is collected from participants in the Exercise alone and CBT+EXER groups during the intervention period.

*Glycated Hemoglobin (A1c)* is measured using O'Bleness Memorial Hospital Laboratory (CLIA certified), West Vrgnia University Hospitals Laboratories (CLIA certified, CAP accredited) and Indiana University Pathology Laboratory (CLIA certified) glycated hemoglobin data from whole blood samples using the measurement of glycated fractions of Hemoglobin A which reflects the glucose level in the blood over a two month time span. The reference range for A1c samples is 4.3-5.7% (23-29 mmol/mol).A1c is the primary diabetes outcome variable.

*Glucose* is drawn at each assessment time point in conjunction with fasting blood lipids and is analyzed by O'Bleness Memorial Hospital Laboratory (CLIA certified), West Virginia University Hospitals Laboratories (CLIA certified, CAP accredited) and Indiana University using CDC-certified and standard enzymatic and precipitation methodology.

*Blood Lipid Profile* is analyzed by O'Bleness Memorial Hospital Laboratory (CLIA certified), West Virginia University Hospitals Laboratories (CLIA certified, CAP accredited) and Indiana University using CDC-certified and standard enzymatic and precipitation methodology for the lipid profile: HDL-C, total cholesterol, LDL-C (direct), and triglycerides.

**Copyright:** © 2015 de Groot M, et al. This is an open-access article distributed under the terms of the Creative Commons Attribution License, which permits unrestricted use, distribution, and reproduction in any medium, provided the original author and source are credited.

*Self-Monitored Blood Glucose (SMBG)* data is gathered from Bayer Contour USB glucomonitors by participants for 1 week following each assessment visit. Individuals randomized to either the Exercise alone or CBT+EXER group are provided with the meter and test strips throughout the intervention period. Data is downloaded and stored throughout the intervention period. Glucometers were donated by Bayer, Inc.

*Six-Minute Walk Test (6MWT)* is used to measure functional exercise status and to estimate aerobic capacity at each assessment visit. The test is performed indoors on a flat, hardened surface. Participants are instructed to cover as much distance as possible within six minutes by walking without running. Total distance covered is measured in meters with a distance measuring wheel [46]. Heart rate, blood pressure and patient symptoms are monitored during the 6MWT.

*Resting, Exercise and Recovery Blood Pressures* is measured via auscultation with a calibrated sphygmomanometer at rest before and after the 6-minute walk test at each assessment time point. Blood pressure may be measured at peak performance by fitness directors during the exercise intervention in conjunction with Borg exertion ratings for clinical monitoring purposes and adherence to the exercise prescription [47].

*Anthropometric measurements* are taken by the project coordinator at each assessment period to estimate body composition and regional adipose distribution. BMI is calculated from height and weight. Height and weight are measured on a Detecto Physician's Scale with stadiometer to the nearest half inch and weight to the pound. Waist circumference (girth) is assessed with a constant tension tape measure (Gulick tape) at the narrowest portion of the torso, while a hip measurement will be made at the maximum posterior extension of the buttocks. Waist to hip ratio is calculated [48].

**Medical History:** Past medical history data is gathered via interview by the project coordinator at each site for T2DM-specific physiologic characteristics such as T2DM duration, height, weight, prescribed diabetes treatment regimen, medical contraindications for participation, accuracy of diagnoses, and number and severity of T2DM complications.

**Medical Status Review:** Changes in medical status are monitored by the project coordinator at each assessment visit for T2DM-specific physiologic effects of exercise on glucose control, changes in medications caused by the intervention, as well as parameters such as height, weight, and number and severity of T2DM complications.
